# Comparison of norovirus genogroup I, II and IV seroprevalence among children in the Netherlands, 1963, 1983 and 2006

**DOI:** 10.1099/jgv.0.000533

**Published:** 2016-09

**Authors:** Janko van Beek, Miranda de Graaf, Ming Xia, Xi Jiang, Jan Vinjé, Mathias Beersma, Erwin de Bruin, David van de Vijver, Melle Holwerda, Marlies van Houten, Annemarie M. Buisman, Rob van Binnendijk, Albert D. M. E. Osterhaus, Fiona van der Klis, Harry Vennema, Marion P. G. Koopmans

**Affiliations:** ^1^​ Department of Viroscience, Erasmus Medical Center, ‘s-Gravendijkwal 230, 3015 CE Rotterdam, The Netherlands; ^2^​ Centre for Infectious Diseases Research, Diagnostics and Screening, National Institute of Public Health and the Environment, Antonie van Leeuwenhoeklaan 9, 3721 MA Bilthoven, The Netherlands; ^3^​ Cincinnati Children's Hospital Medical Center, University of Cincinnati College of Medicine, 3333 Burnet Ave, Cincinnati, OH 45229, USA; ^4^​ Division of Viral Diseases, Centers for Disease Control and Prevention, 1600 Clifton Rd, Atlanta, GA 30333, USA; ^5^​ Pediatric Department, Spaarne Hospital Hoofddorp, Hoofddorp, The Netherlands

**Keywords:** Caliciviridae, norovirus, gastroenteritis, seroprevalence, Netherlands

## Abstract

Noroviruses are a major cause of acute gastroenteritis worldwide and are a genetically diverse group of viruses. Since 2002, an increasing number of norovirus outbreaks have been reported globally, but it is not clear whether this increase has been caused by a higher awareness or reflects the emergence of new genogroup II genotype 4 (GII.4) variants. The hypothesis that norovirus prevalence has increased post-2002 and is related to the emergence of GII.4 is tested in this study. Sera collected from children aged <5 years of three Dutch cross-sectional population based cohorts in 1963, 1983 and 2006/2007 (*n*=143, *n*=130 and *n*=376, respectively) were tested for specific serum IgG by protein array using antigens to GII.4 and a range of other antigens representing norovirus GI, GII and GIV genotypes. The protein array was validated by paired sera of norovirus infected patients and supernatants of B-cell cultures with single epitope specificity. Evidence for norovirus infection was found to be common among Dutch children in each cohort, but the prevalence towards different genotypes changed over time. At the genogroup level, GI seroprevalence decreased significantly between 1963 and 2006/2007, while a significant increase of GII and, in particular, specific antibodies of the genotype GII.4 was detected in the 2006/2007 cohort. There were no children with only GII.4 antibodies in the 1963 cohort. This study shows that the high GII.4 norovirus incidence in very young children is a recent phenomenon. These findings are of importance for vaccine development and trials that are currently focusing mostly on GII.4 viruses.

## Introduction

Noroviruses belong to the family *Caliciviridae* and are a major cause of acute gastroenteritis in outbreaks and sporadic cases for all age groups worldwide (Ahmed *et al.*, 2014). Noroviruses are genetically highly diverse positive-stranded RNA viruses that can be divided into six genogroups (G), with a seventh genogroup that has been recently proposed (Vinjé, 2015). Viruses of GI, GII and GIV are known to cause diarrhoeal disease in humans. The genogroups are further divided into approximately 40 genotypes based on their phylogenetic clustering (Kroneman *et al.*, 2013). The lack of a robust cell culture system for norovirus has hampered the development of serological assays to study the impact of individual norovirus genotypes on the population. To be able to measure the immune response to norovirus infection, ELISA assays based on virus-like particles (VLPs) produced through expression of the viral capsid protein (VP1) have been developed, but these assays cannot distinguish exposure to different genotypes due to high levels of cross-reactivity (Rockx *et al.*, 2005). As a surrogate for virus neutralization assays, assays have been developed to measure antibodies that block the binding of noroviruses to histo-blood group antigens (Harrington *et al.*, 2002). These assays eliminate cross-reactivity observed in ELISA assays with VLPs, but are not suitable for population studies since they are difficult to standardize, time- consuming and need large quantities of serum and VLPs. The VP1 consists of a conserved shell (S) domain and the more variable P domain that contains all immunogenic sites. Upon expression of the norovirus P domain P particles will be formed, which are very stable and immunologically relevant, but which contain less cross-reactive epitopes owing to the absence of the conserved S-domain, as evidenced from comparative immunization studies in mice, will be formed (Tamminen *et al.*, 2012; Tan *et al.*, 2004).

Since the start of norovirus surveillance in the mid-1990s, genogroup II genotype 4 (GII.4) has been the predominant genotype across the globe, responsible for 62 % of outbreaks and the majority of endemic illnesses (Ahmed *et al.*, 2014; Siebenga *et al.*, 2009; Verhoef *et al.*, 2015). The norovirus GII.4 epidemiology has similarities to that of influenza A viruses, with new antigenic variants emerging every 2–3 years that replace the previously established variant and herd immunity as the main evolutionary driving force (Lindesmith *et al.*, 2012; Siebenga *et al.*, 2007b). Since the mid-1990s, six GII.4 variants with pandemic spread have been recognized: US95/96, Farmington Hills 2002, Hunter 2004, Den Haag 2006b, New Orleans 2009 and Sydney 2012 (Eden *et al.*, 2013). Little is known about the genetic diversity of the norovirus before the mid-1990s since molecular techniques were not available then and historical faecal collections are exceedingly rare. To our knowledge only one study has looked at the GII.4 molecular epidemiology before 1996 and has found GII.4 only in 9 of 48 (18.8 %) faecal samples collected between 1974 and 1991 (Boon *et al.*, 2011). Since the appearance of the GII.4 Farmington Hills 2002 variant, an increasing number of norovirus outbreaks have been reported compared with previous years, but it is not clear whether this increase has been caused by a higher awareness or is an effect of the advancement of the emergence of antigenic drift variants of GII.4 or other evolutionary effects leading to increased fitness of these viruses at the population level (Lopman *et al.*, 2004; Siebenga *et al.*, 2010). Therefore, we wanted to test the hypothesis that the emergence of predominant GII.4 viruses have been driving the increased norovirus burden since 2002. Sera from children under the age of 5 years were selected from three cross-sectional population-based serum cohorts collected in 1963, 1983 and 2006/2007 and tested with a novel multiplex protein array to detect antibody responses to individual norovirus genotypes. Sera of young children were chosen to measure the impact of exposure to noroviruses in the first years of life. The protein array was validated using polyclonal rabbit sera, pre- and post-sera of norovirus infected individuals, and supernatants of B-cell cultures with single epitope specificity.

## Results

### Array specificity

A novel multiplex norovirus P particle protein array was developed to help measure norovirus genotype-specific antibodies. First, the specificity of the newly developed array was confirmed using polyclonal sera of two rabbits immunized with VP1 protein from GII.4 and GIV, respectively. Both the GII.4 and GIV rabbit sera reacted with high signal with the homologous antigen without significant cross-reaction with the heterologous antigens (data not shown). Next, we tested pre- and post-infection sera of four norovirus reverse transcriptase (RT-PCR) confirmed patients infected with GII.4 Den Haag 2006b, GII.4 New Orleans 2009, GII.3 and GI.6, respectively (Table 1, Figure S1, available in the online Supplementary Material). All patients showed a more than fourfold increase with the homologous antigen showing that P particles in the array platform remain intact and are recognized by norovirus antibodies in patients. The pre-infection sera of three patients (Table 1 patients B, C and D) already showed reactivity, which was most likely caused by previous infections as expected, since noroviruses are one of the most common infections during childhood. Antibody responses in the three GII infected patients (Table 1 patients A, B and C) were exclusively observed in the case of GII antigens, in particular, the homologous antigen, with low levels of cross-reactivity or boosting of previous infections with heterologous GII antigens. The serum sample of the GI.6 infected patient (Table 1 patient D) showed a specific GI.6 reaction and did not bind to the heterologous GI and GII antigens.

**Table 1.** Antibody titres (95 % confidence interval) in pre- and post-infection sera

Patient	Pre/post-infection	Days relative to day of onset	Infecting genotype	GI.1	GI.2	GI.6	GI.8	GII.3	GII.4	GII.9
A	Pre	−32	Unknown history	20	20	20	20	20	20	20
A	Post	52	GII.4 Den Haag 2006b	20	20	20	20	411 (331–491)	5120	909 (710–1107)
B	Pre	−3	Unknown history	20	20	316 (281–352)	20	20	20	20
B	Post	46	GII.4 New Orleans 2009	20	20	145 (120–171)	20	86 (72–101)	5120	554 (495–613)
C	Pre	−1	Unknown history	20	20	20	20	136 (120–151)	20	20
C	Post	13	GII.3	20	20	20	20	2481 (2058–2904)	191 (165–217)	249 (223–275)
D	Pre	−4	Unknown history	125 (93–158)	87 (81–94)	100 (90– 110)	20	20	250 (222–278)	63 (56–70)
D	Post	46	GI.6	20	20	553 (491–615)	20	20	127 (105–149)	20

### Quality control

Using a positive control serum, consisting of pooled human sera, we determined that the average intra-assay coefficient of variation (CV) for antibodies to individual P particle antigens was 9.0 % (range 6.2–13.7 %) and average CV inter-assay variation 25.7 % (range 16.9–36.0 %). The VLP GIV was not included in this analysis since the human control serum was not reactive to this antigen. The potential degradation of antibodies over time was monitored by comparing the magnitude of titres among cohorts. High antibody titres against individual antigens were observed in all three cohorts, indicating that the serum antibodies were not degraded (Fig. S2).

### Potential cross-reactivity of antibodies towards the antigens on the array

**Fig. 1.**
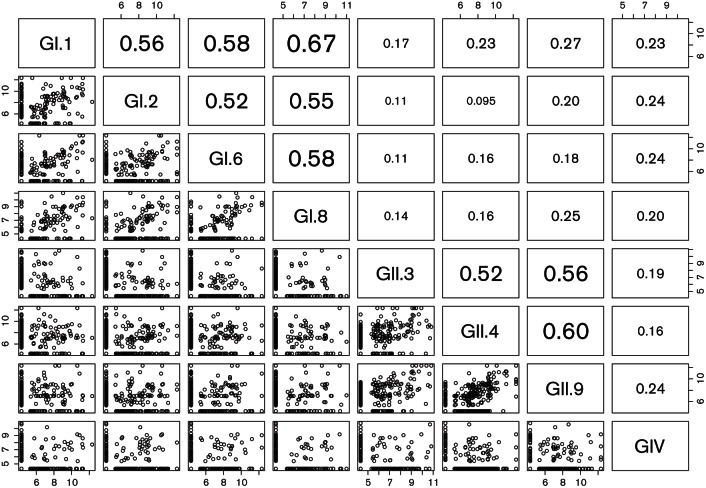
Multiple scatterplot log-transformed titres of children <5 years (*n*=649) to determine potential cross-reactive patterns among P particle antigens on the protein array platform. In the top right triangle Spearman correlation coefficients are plotted between each antigen pair, with significant numbers being highlighted by enlargement.

Sera of children below 5 years of age (*n*=649) were assessed for potential cross-reactivity patterns using the Spearman correlation coefficient and plotting of log-transformed titres between each possible antigen pair (Fig. 1). Antibodies directed to antigens belonging to different genogroups typically did not show significant cross-reactivity in this age group, with a low correlation coefficient ranging from 0.01 to 0.27. Within a genogroup, some cross-genotype antibody reactivity was observed with correlation coefficients for heterotypic antibody reactivity ranging between 0.52 and 0.67. This pattern was not consistent among the sera, indicating that cross-reactive patterns are complex, reflecting large individual variability rather than a primary technical cause.

### B-cell supernatants with single epitope specificity

The observation that the sera of some of the young children were reactive to multiple antigens raised the question whether these children were already infected with multiple norovirus genotypes, or their sera contained cross-reactive antibodies, or both. Paired sera of 40 children obtained at 9 and 10 years of age were screened on the protein array for the presence of broadly reacting serum antibody profiles. Two donors with a broad serum profile were selected and B-cells of these donors were polyclonally stimulated to become antibody secreting cells (ASC). Limiting dilution was carried out to create multiple cultures with single epitope specificity. B-cell supernatants were tested on the array to dissect the serological profiles of both donors, using limiting dilution to select for single B-cell clones per well (Baas *et al.*, 2013; Pinna *et al.*, 2009). Bulk cultures were first tested and all these cultures reacted with one or multiple antigens, confirming the serological reactivity observed in the sera of these children. For donor 1, only 2 of 14 (14.3 %) pools containing 8000 cells each were positive and both positive clones had single genotype specificity to genotype GII.3 (Table 2). Donor 2 did not meet the clonal criteria as 9 of 16 (56 %) pools showed positive reaction to one or multiple norovirus antigens. Some supernatants showed broad GI reactivity, suggesting either the presence of a broadly reactive epitope, or mixed B-cell populations. Subsequent testing of individual supernatants' pool 12 and 13 confirmed reactivity to multiple genotypes within GI, as only 1 of 8 cultures harboured reactivity to norovirus antigens, indicating that these reactivities were caused by a broadly reactive epitope. However, in both donors, multiple clones with single genotype specificity were also obtained, with GII.3 the only reactive supernatant in donor 1, and GI.6- and GII.4-specific supernatants in donor 2, showing that technically, the array can measure genotype-specific human antibodies.

**Table 2.** B-cell supernatant profiles of two donors with a broadly reacting serum profile Symbols −, +, ++, +++ represent, respectively, a fluorescent signal of 0–500, 501–5 000, 5 001–30 000, >30 000 RFU.

	Donor	Cells	GI.1	GI.2	GI.6	GI.8	GII.3	GII.4	GII.9
Pools 1–12	1	8000	−	−	−	−	−	−	−
Pool 13	1	8000	−	−	−	−	+	−	−
Pool 14	1	8000	−	−	−	−	+++	−	−
Pools 1–7	2	4000	−	−	−	−	−	−	−
Pool 8	2	4000	−	−	−	−	−	++	−
Pool 9	2	4000	−	+	−	+	−	−	−
Pool 10	2	4000	−	+	−	−	−	−	−
Pool 11	2	4000	−	−	+	−	−	−	−
Pool 12	2	4000	+++	+++	+++	++	−	−	−
Pool 12 Culture 1–7	2	500	−	−	−	−	−	−	−
Pool 12 Culture 8	2	500	+++	+++	+++	++	−	−	−
Pool 13	2	4000	−	+++	++	−	−	−	−
Pool 13 Culture 1–7	2	500	−	−	−	−	−	−	−
Pool 13 Culture 8	2	500	−	+++	+	−	−	−	−
Pool 14	2	4000	−	−	−	−	−	+++	−
Pool 15	2	4000	−	+	−	−	++	−	−
Pool 16	2	4000	++	++	++	++	−	−	−

### Seroprevalence

Next, the seroprevalence against different norovirus genotypes in children <5 years (*n*=649) was determined. Based on a pilot experiment with the sera of children <1 year of age, a cut-off for positive samples was set at titre 40 (data not shown). The overall norovirus IgG seroprevalence has not significantly changed over time with 69.2 %, 70.0 % and 65.4 % being seropositive for at least one genotype in 1963, 1983 and 2006/2007, respectively (Table 3, chi-squared test for trend, *P* value=0.3307). In children below 1 year of age, seroprevalence was already at a high level in 1963 and 1983 (72.0 % and 70.0 %, respectively), likely reflecting maternal antibodies. In the 2006/2007 cohort the seroprevalence for this age category was significantly lower (42.7 %). More than half of the children were already seropositive by the second year of life, increasing to 85.3, 75.9 and 85.0 % at age 4 in 1963, 1983 and 2006/2007, respectively.

**Table 3.** Age stratified number of sera tested and percentage of sera with reactivity to any norovirus antigen

	1963		1983		2006/2007	
Age group(years)	*n* sera tested	Sera norovirus positive (%)	*n* sera tested	Sera norovirus positive (%)	*n* sera tested	Sera norovirus positive (%)
<1	25	72.0	20	70.0	110	42.7
1 to <2	32	50.0	23	56.5	57	64.9
2 to <3	25	72.0	34	67.6	74	63.5
3 to <4	27	66.7	24	79.2	75	85.3
4 to <5	34	85.3	29	75.9	60	85.0
Total	143	69.2	130	70.0	376	65.4

**Fig. 2.**
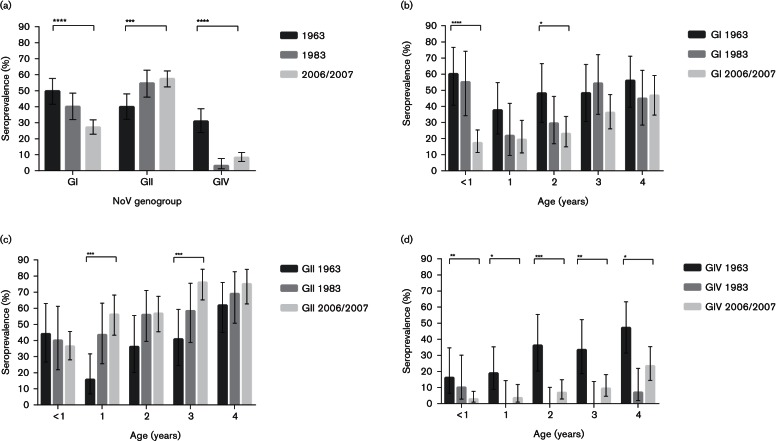
Comparison of norovirus seroprevalence (*n*=649) stratified to cohort and genogroup (a). Age-related seroprevalence (*n*=649) stratified to cohort for norovirus genogroup I (b), genogroup II (c) and genogroup IV (d). Error bars indicate binomial proportion confidence intervals (Wilson score interval). Brackets above bars show significance level as determined by chi-squared test for trend. No bracket =*P*>0.05, *P≤0.05, **P≤0.01, ***P≤0.001 and ****P ≤0.0001.

**Fig. 3.**
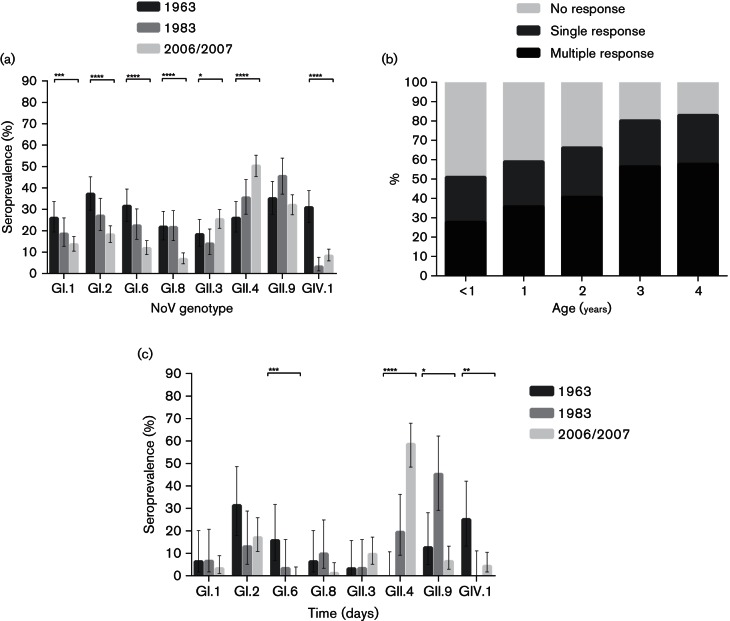
Comparison of norovirus seroprevalence (*n*=649) stratified to cohort and genotype (a). Age-related broadening of the norovirus immune response (*n*=649) (b). Comparison of norovirus seroprevalence in sera (*n*=157) with single response stratified to cohort and genotype (c). Error bars indicate binomial proportion confidence intervals (Wilson score interval). Brackets above bars show significance level as determined by chi-squared test for trend. No bracket = *P*>0.05, *P≤0.05, **P≤0.01, ***P≤0.001 and ****P≤0.0001.

Interestingly, however, substantial differences were observed when breaking the reactivity down to genogroups and genotypes. At the genogroup level, GI seroprevalence dropped over time while the prevalence of GII antibodies increased (Fig. 2a). GIV prevalence was much higher in the first cohort, with 30.8 % in 1963 compared with 3.1 % and 8.2 % in 1983 and 2006/2007. Stratified by age, the highest GI seroprevalence decrease was seen among children of age <1 year and 2 years, while the largest GII increase was seen among children of 1 and 3 years (Fig. 2b, c). Seroprevalence for GIV was significantly higher in all age years in 1963 compared with cohort 1983 and 2006/2007 (Fig. 2d). All four tested GI genotypes showed a significant reduction in seroprevalence over time, while the increase in GII antibodies was primarily caused by an increased reactivity to GII.3 and GII.4 antigens (Fig. 3a). The antigen with highest seroreactivity was GI.2 in 1963, GII.9 in 1983 and GII.4 in 2006/2007.

### Broadening of immune response by increasing age

The multiplex composition of the protein array platform enabled the simultaneous detection of norovirus antibodies to various genotypes and allowed us to measure the age-related broadening of the norovirus immune response (Fig. 3b). Using a cut-off of 40, the sera were stratified into three groups: sera without reactivity to any norovirus antigen, sera with reactivity to a single antigen only and sera with multiple reactivity. A clear age-related increase in sera with multiple responses was observed, while the proportion of sera with monospecific responses remained stable (*P* value chi-squared test for trend among multiple, monospecific and non-responders, respectively: <0.0001, <0.6981 and <0.0001).

### Monospecific responses

To eliminate false positives due to the presence of broadly reactive antibodies, 157 sera from children with antibodies limited to a single antigen were analysed separately (Table 4, Fig. 3c). Interestingly, GII.4 antibodies were not detected as a unique profile in the 1963 cohort, whereas they constituted 19.4 % of monospecific sera in 1983 and were predominant with 58.5 % in 2006/2007 indicating a dramatic increase of antibodies to GII.4, or a closely related genotype, over time. Furthermore, among antigens tested, GI.2 was predominant in 1963 and GII.9 was predominant in 1983.

**Table 4.** Genotype distribution within positive sera with single antigen response

Cohort	GI.1		GI.2		GI.6		GI.8		GII.3		GII.4		GII.9		GIV.1		Total	
	*n*	%	*n*	%	*n*	%	*n*	%	*n*	%	*n*	%	*n*	%	*n*	%	*n*	%
1963	2	6.3	10	31.3	5	15.6	2	6.3	1	3.1	0	0.0	4	12.5	8	25.0	32	100
1983	2	6.5	4	12.9	1	3.2	3	9.7	1	3.2	6	19.4	14	45.2	0	0.0	31	100
2006/2007	3	3.2	16	17.0	0	0.0	1	1.1	9	9.6	55	58.5	6	6.4	4	4.3	94	100

## Discussion

In this cross-sectional population study the norovirus seroprevalence was investigated among children <5 years of age from whom serum was collected in 1963, 1983 or 2006/2007. The results show that norovirus has been a common and widespread infection among Dutch children, at least since 1963, with no indication of an increase in the overall seroprevalence. This confirms prior studies using ELISA or related assays based on recombinant VLPs in China, Finland, India, Japan, Korea, Spain and the UK, although quite some variation in seroprevalence rates were observed by geographic location and sample year, which may be due, in part, to a lack of assay standardization (Carmona-Vicente *et al.*, 2015; Kobayashi *et al.*, 2009; Menon *et al.*, 2013; Nurminen *et al.*, 2011; Son *et al.*, 2013).

It has been hypothesized that the emergence of GII.4 has resulted in a large increase of norovirus outbreaks since 2002 (Lopman *et al.*, 2004; Siebenga *et al.*, 2010). Therefore, we assessed exposure to individual norovirus genotypes using a novel protein array. The validation experiments showed that a person’s serological profile may contain a mixture of genotype-specific and broadly-reactive antibodies with cross genogroup reactivity, although clear discriminatory serological profiles were obtained on looking at post-infection sera. This pattern is in agreement with the higher correlations found between antibodies and antigens of the same genogroup compared to that with antigens from different genogroups. Based on these observations, we concluded that the study design allows conclusions based on antibody trends by genogroup and genotype, although for the latter, the potential for cross-reactive antibodies needs to be considered when interpreting the results.

Our study shows that – despite similar overall seroprevalence – the exposure histories shifted drastically over the period studied, and that the seroprevalence of GII.4 has indeed significantly increased over the last decades, with concomitant reduction in exposure to GI and GIV noroviruses. In theory, the low GII.4 seroprevalence in the 1963 and 1983 cohort could be caused by a mismatch between circulating GII.4 variants and the antigen used in our assay (GII.4 US95/96 antigen). This is however unlikely, as sera from patients infected with recent GII.4 variants bound efficiently to the GII.4 US95/96 variant antigen. Therefore, we conclude that the most likely explanation for the observed shift in norovirus exposure is that it reflects true epidemiological changes. The absence of sera from the 1963 cohort that only reacted to GII.4 suggests that either GII.4 viruses were not circulating, or that exposure to this genotype occurred at an older age.

Based on these data alone, we cannot draw conclusions on the clinical impact of these shifts in exposure. All norovirus genotypes are associated with diarrhoeal disease, but GII.4 strains are believed to be more transmissible in healthcare settings, also cause more severe disease and are strongly associated with the seasonal patterns in reporting (Desai *et al.*, 2012; Siebenga *et al.*, 2007a). The combination of the GII.4 variant pandemics, the association between GII.4 and more severe symptoms, and the high GII.4 infection rates among very young children shown in this study, could potentially explain the increase of norovirus disease reports since 2002. A potential bias in this study is that young children have not yet build up an immune response against norovirus and therefore are likely susceptible to any strain with matching receptor specificity, while adults have already been exposed multiple times to norovirus. Conclusions based on the seroprevalence among young children can therefore not be one-to-one translated to adults.

We also observed broadening of the antibody profile in young children with increasing age, and reactivity to antigens representing genotypes that are rarely found in the Dutch national molecular surveillance. While we cannot rule out that this broadening of antibody profile reflects asymptomatic infections, another explanation is that these effects are caused by high-affinity antibodies directed against shared epitopes on the P domain of VP1, produced by B-cells that undergo somatic hypermutation and clonal selection upon multiple exposures to different genotypes. Although most conserved epitopes are found on the S domain of VP1, the B-cell supernatants in this study and studies with mAbs have shown that the P domain contains conserved epitopes as well (Shiota *et al.*, 2007). An important question is whether such antibodies influence the susceptibility to subsequent infection, as has been observed with the discovery of broadly reactive non-haemagglutination inhibition antibodies in influenza A (Wrammert *et al.*, 2011).

The shifting of antibody profiles for the different cohorts shows that a GI genotype was dominant in 1963 and a GII, other than GII.4, in 1983, which may indicate that multiple dominant genotype switches have taken place before the predominance of GII.4 viruses. Although the overall norovirus seroprevalence did not change over the years in this study, it seems likely that genotype replacements may have concurred with a temporary increase in outbreak activity as is seen with GII.4 variant replacements (van Beek *et al.*, 2013). Further studies are warranted to measure the effect of changes in the norovirus epidemiology on the norovirus incidence in the population and related costs for the society.

The first norovirus vaccine candidates have been tested in clinical trials and have been shown to curb severity of disease after a challenge with a homologous strain, which may be helpful to reduce hospital admissions in vulnerable patient populations and associated costs (Bernstein *et al.*, 2015). Field efficacy, cross-protection to drifted variants and heterologous genotypes, efficacy in various age groups and duration of protection needs to be evaluated in future studies. Most importantly, however, our data suggests that special attention needs to be paid to the potential for updating the vaccine composition following changes in the norovirus epidemiology. Current vaccine candidates are based on the norovirus epidemiology associated with the dominance of GII.4, but with the recent emergence of GII.17 in Asia, the vaccine candidates may need to be updated (de Graaf *et al.*, 2015). Continued surveillance and a better understanding of norovirus epidemiology is essential knowledge for an optimal vaccine design.

## Methods

### Antigens.

Norovirus P particles were used as antigens since they antigenically resemble native virions and can be produced in *Escherichia coli *expression systems at relatively low costs as described (Jiang *et al.*, 1992; Tan & Jiang, 2005). Furthermore, P particles only contain the highly variable protruding (P) domain of the viral capsid protein (VP1), lack the more conserved S domain and, therefore, contain less cross-reactive epitopes, as evidenced by comparative immunization studies in mice (Tamminen *et al.*, 2012). A GIV.1 VLP produced in insect cells was added to include antigens from all human genogroups. Since GIV is genetically not closely related to other human genogroups we did not expect cross-reactivity between these VLP and the P particles representing genotypes of GI and GII. We selected antigens representing common and rare genotypes as detected by the Noronet sequence database (Table S1, available in the online Supplementary Material) (http://www.noronet.nl).

### Norovirus protein microarrays for multiplex serology.

Purified P particles and VLPs were diluted in protein array buffer (Maine Manufacturing) and protease inhibitor (BioVision), with final concentration of 1 mg ml^−1^ (determined by checkerboard titration, data not shown). Proteins were spotted in triplicate with two 333 pL spots onto 64-pad nitrocellulose-coated slides (Oncyte avid, Grace bio-labs) using a non-contact Piezorray spotter (PerkinElmer) as described previously (Koopmans *et al.*, 2012). Slides were incubated with Blotto blocking buffer (Thermo Fisher Scientific) to avoid non-specific nitrocellulose binding, and subsequently with serial fourfold diluted human sera starting at a 1 : 40 dilution. Rabbit sera and B-cell supernatants were tested at a single dilution (1 : 20 for rabbit sera, 1 : 8 for B-cell supernatant pools and 1 : 4 for individual cultures). After washing, slides were incubated with goat anti-human IgG or anti-rabbit IgG (Fc-fragment specific), conjugated with Alexa Fluor 647 fluorescent dye (Jackson Immuno Research). Bound dye was quantified using a ScanArray Gx Plus microarray scanner (PerkinElmer).

### Assay validation samples.

Two rabbit polyclonal sera from animals immunized with recombinant norovirus GII.4 Den Haag 2006b or GIV VP1 protein (Immune Technology) were tested on the protein array to confirm that antigens remain intact on the platform and to test homologous versus heterologous antigen reactivity. Pre- and post-infection sera of RT-PCR confirmed norovirus patients were used for assay validation. These patients were infected with GII.4 Den Haag 2006b, GII.4 New Orleans 2009, GII.3 or GI.6, respectively (ages 5, 47, 17 and 12 years). Use of the sera for assay validation was approved by the Erasmus MC medical ethical committee (MEC2013-082). Further validation of the array platform was performed by using sera (*n*=40, storage at −20 °C) and PBMCs (storage at −135 °C) obtained from two donors (10 years) who were sampled for a study on the memory immunity to *Bordetella pertussis* (ISRCTN64117538).

### Quality control.

The intra- and inter-assay variation was monitored by testing a serial diluted positive control serum consisting of pooled human sera reacting with high norovirus titre to antigens belonging to genogroup I and II. Samples tested on slides with a positive control deviating more than one twofold dilution step from the geometric mean titre were rejected from analysis. For the intra-assay variation, the positive control serum was tested 16 times on a single slide and to determine the inter-assay variation, the control serum was tested 44 times on multiple slides within 13 weeks. The quality of the GIV VLP was tested with a rabbit control serum (data not shown).

### B-cell supernatants with single epitope specificity.

B-cell supernatants with single epitope specificity were used to test the specificity of the protein array platform. B-cells were isolated, stimulated and cultured in a limiting dilution assay with a slightly adjusted protocol as described before (Baas *et al.*, 2013). Briefly, PBMC were isolated within 24 h after venepuncture and stored at −135 °C for further use. The EasySep™ Human CD19 positive selection kit and EasySep magnet (Stemcell Technologies) were used to isolate the B-cells from the PBMC population. Purified B-cells were counted and re-suspended in 96-well round bottom tissue culture plates with each well containing 500, 1000 or 4000 B-cells. Gamma irradiated CD40L-expressing murine ﬁbroblast L cells were added in a concentration of 500 cells per well and 3 µg ml^−1^ CpG ODN2006 (Isogen Life Sciences), 10 ng ml^−1^IL-2 (Miltenyi Biotec) and 10 ng ml^−1^ IL-10 (BD Pharmingen) was added to promote cell division and ASC outgrowth. After 5 days of incubation at 37 °C culture medium was refreshed and cytokines were replaced by 10 ng ml^−1^ IL-2 and 10 ng ml^−1^ IL-21 (Invitrogen) to promote antibody production. Supernatants were harvested after 11–12 days, stored at −20 °C, and *in vitro* IgG production was tested by total IgG ELISA (data not shown). Supernatants were subsequently tested against multiple antigens representing GI and GII genotypes on the protein array platform.

### Study samples.

A total of 649 serum samples from children <5 years of age collected in 1963, 1983 and 2006/2007 were included in the study, with *n*=143, *n*=130 and *n*=376, respectively (Table 3). The sera collected in 1963 and 1983 were obtained from a Dutch historical anonymous collection of serum samples, which had been collected for diagnostic purposes (not specifically for acute gastroenteritis), and stored at −20 °C since then at Erasmus MC, Rotterdam (Masurel & Mulder, 1966). The sera collected between February 2006 and June 2007 were obtained from a Dutch population-based cross-sectional serosurvey (van der Klis *et al.*, 2009). The study was approved by the Medical Ethics Committee, Almere (ISRCTN 20164309).

### Data and statistical analysis.

Serum titres were computed by fitting a four-parameter log-logistic curve to 12 luminescence readouts (four dilutions, each antigen tested in triplicate), using the point of inflection as titre, as described (Koopmans *et al.*, 2012). A fixed minimum fluorescent signal of 3000 was chosen to reduce background reactivity and a fluorescent signal of 65 535 was used as fixed maximum readout. Titres below the minimum dilution were set to a value half of the reciprocal of the minimal dilution and titres above the highest dilution were set to a value of two times the reciprocal of the highest serum dilution. Data of B-cell supernatants is shown as fluorescent signal (relative fluorescence units, RFU) with the background signal subtracted since the supernatants were tested at a single dilution. Data analyses and statistical analysis were performed using R version 3.0.3. The chi-squared test for trend was performed to examine differences in seroprevalence rates between age groups and cohorts. A *P* value ≤0.05 was considered to be significant. The Spearman correlation coefficient was used to assess potential cross-reactivity patterns between antibody titres detected against multiple norovirus genotypes.

## Supplementary Data

1128 Supplementary File 1

## References

[R2] Ahmed S. M. Hall A. J. Robinson A. E. Verhoef L. Premkumar P. Parashar U. D. Koopmans M. Lopman B. A.(2014). Global prevalence of norovirus in cases of gastroenteritis: a systematic review and meta-analysis. Lancet Infect Dis 14 725–730. 10.1016/S1473-3099(14)70767-4 24981041 PMC8006533

[R3] Baas D. C. Koopmans M. P. de Bruin E. ten Hulscher H. Buisman A. M. Hendrikx L. H. van Beek J. Godeke G. J. Reimerink J. van Binnendijk R. S.(2013). Detection of influenza A virus homo- and heterosubtype-specific memory B-cells using a novel protein microarray-based analysis tool. J Med Virol 85 899–909. 10.1002/jmv.23535 23508915

[R4] Bernstein D. Atmar R. L. Lyon G. M. Treanor J. J. Chen W. H. Jiang X. Vinjé J. Gregoricus N. Frenck R. W., Jr.(2015). Norovirus vaccine against experimental human GII.4 virus illness: a challenge study in healthy adults. J Infect Dis 211 870–878. 10.1093/infdis/jiu497 25210140 PMC5914500

[R5] Boon D. Mahar J. E. Abente E. J. Kirkwood C. D. Purcell R. H. Kapikian A. Z. Green K. Y. Bok K.(2011). Comparative evolution of GII.3 and GII.4 norovirus over a 31-year period. J Virol 85 8656–8666. 10.1128/JVI.00472-11 21715504 PMC3165818

[R6] Carmona-Vicente N. Fernández-Jiménez M. Ribes J. M. Téllez-Castillo C. J. Khodayar-Pardo P. Rodríguez-Diaz J. Buesa J.(2015). Norovirus infections and seroprevalence of genotype GII.4-specific antibodies in a Spanish population. J Med Virol 87 675–682. 10.1002/jmv.24112 25655810

[R32] de Graaf M. van Beek J. Vennema H. Podkolzin A. T. Hewitt J. Bucardo F. Templeton K. Mans J. Nordgren J.(2015). Emergence of a novel GII.17 norovirus – end of the GII.4 era? Euro Surveill 20 21178. 10.2807/1560-7917.ES2015.20.26.21178 26159308 PMC5921880

[R7] Desai R. Hembree C. D. Handel A. Matthews J. E. Dickey B. W. McDonald S. Hall A. J. Parashar U. D. Leon J. S. Lopman B.(2012). Severe outcomes are associated with genogroup 2 genotype 4 norovirus outbreaks: a systematic literature review. Clin Infect Dis 55 189–193. 10.1093/cid/cis372 22491335 PMC3491774

[R8] Eden J. S. Tanaka M. M. Boni M. F. Rawlinson W. D. White P. A.(2013). Recombination within the pandemic norovirus GII.4 lineage. J Virol 87 6270–6282. 10.1128/JVI.03464-12 23536665 PMC3648122

[R9] Harrington P. R. Lindesmith L. Yount B. Moe C. L. Baric R. S.(2002). Binding of Norwalk virus-like particles to ABH histo-blood group antigens is blocked by antisera from infected human volunteers or experimentally vaccinated mice. J Virol 76 12335–12343. 10.1128/JVI.76.23.12335-12343.2002 12414974 PMC136916

[R10] Jiang X. Wang M. Graham D. Y. Estes M. K.(1992). Expression, self-assembly, and antigenicity of the Norwalk virus capsid protein. J Virol 66 6527–6532. 1328679 10.1128/jvi.66.11.6527-6532.1992 PMC240146

[R11] Kobayashi S. Fujiwara N. Takeda N. Minagawa H.(2009). Seroepidemiological study of norovirus infection in Aichi Prefecture, Japan. Microbiol Immunol 53 356–359. 10.1111/j.1348-0421.2009.00132.x 19493204

[R12] Koopmans M. de Bruin E. Godeke G. J. Friesema I. van Gageldonk R. Schipper M. Meijer A. van Binnendijk R. Rimmelzwaan G. F.(2012). Profiling of humoral immune responses to influenza viruses by using protein microarray. Clin Microbiol Infect 18 797–807. 10.1111/j.1469-0691.2011.03701.x 22212116

[R13] Kroneman A. Vega E. Vennema H. Vinjé J. White P. A. Hansman G. Green K. Martella V. Katayama K. Koopmans M.(2013). Proposal for a unified norovirus nomenclature and genotyping. Arch Virol 158 2059–2068. 10.1007/s00705-013-1708-5 23615870 PMC5570552

[R14] Lindesmith L. C. Beltramello M. Donaldson E. F. Corti D. Swanstrom J. Debbink K. Lanzavecchia A. Baric R. S.(2012). Immunogenetic mechanisms driving norovirus GII.4 antigenic variation. PLoS Pathog 8 e1002705. 10.1371/journal.ppat.1002705 22615565 PMC3355092

[R15] Lopman B. Vennema H. Kohli E. Pothier P. Sanchez A. Negredo A. Buesa J. Schreier E. Reacher M.(2004). Increase in viral gastroenteritis outbreaks in Europe and epidemic spread of new norovirus variant. Lancet 363 682–688. 10.1016/S0140-6736(04)15641-9 15001325

[R16] Masurel N. Mulder J.(1966). Studies on the content of antibodies for equine influenza viruses in human sera. Bull World Health Organ 34 885–893. 5296537 PMC2476048

[R17] Menon V. K. George S. Aladin F. Nawaz S. Sarkar R. Lopman B. Gray J. J. Gomara M. Kang G.(2013). Comparison of age-stratified seroprevalence of antibodies against norovirus GII in India and the United Kingdom. PLoS One 8 e56239. 10.1371/journal.pone.0056239 23437102 PMC3578856

[R18] Nurminen K. Blazevic V. Huhti L. Räsänen S. Koho T. Hytönen V. P. Vesikari T.(2011). Prevalence of norovirus GII-4 antibodies in Finnish children. J Med Virol 83 525–531. 10.1002/jmv.21990 21264875

[R19] Pinna D. Corti D. Jarrossay D. Sallusto F. Lanzavecchia A.(2009). Clonal dissection of the human memory B-cell repertoire following infection and vaccination. Eur J Immunol 39 1260–1270. 10.1002/eji.200839129 19404981 PMC3864550

[R20] Rockx B. Baric R. S. de Grijs I. Duizer E. Koopmans M. P.(2005). Characterization of the homo- and heterotypic immune responses after natural norovirus infection. J Med Virol 77 439–446. 10.1002/jmv.20473 16173019

[R21] Shiota T. Okame M. Takanashi S. Khamrin P. Takagi M. Satou K. Masuoka Y. Yagyu F. Shimizu Y.(2007). Characterization of a broadly reactive monoclonal antibody against norovirus genogroups I and II: recognition of a novel conformational epitope. J Virol 81 12298–12306. 10.1128/JVI.00891-07 17855545 PMC2168978

[R22] Siebenga J. J. Vennema H. Duizer E. Koopmans M. P.(2007a). Gastroenteritis caused by norovirus GGII.4, The Netherlands, 1994–2005. Emerg Infect Dis 13 144–146. 10.3201/eid1301.060800 17370531 PMC2913659

[R23] Siebenga J. J. Vennema H. Renckens B. de Bruin E. van der Veer B. Siezen R. J. Koopmans M.(2007b). Epochal evolution of GGII.4 norovirus capsid proteins from 1995 to 2006. J Virol 81 9932–9941. 10.1128/JVI.00674-07 17609280 PMC2045401

[R1] Siebenga J. J. Vennema H. Zheng D. P. Vinjé J. Lee B. E. Pang X. L. Ho E. C. Lim W. Choudekar A.(2009). Norovirus illness is a global problem: emergence and spread of norovirus GII.4 variants, 2001–2007. J Infect Dis 200 802–812. 10.1086/605127 19627248

[R24] Siebenga J. J. Lemey P. Kosakovsky Pond S. L. Rambaut A. Vennema H. Koopmans M.(2010). Phylodynamic reconstruction reveals norovirus GII.4 epidemic expansions and their molecular determinants. PLoS Pathog 6 e1000884. 10.1371/journal.ppat.1000884 20463813 PMC2865530

[R25] Son H. Jeong H. S. Cho M. Lee J. Lee H. Yoon K. Jeong A. Y. Jung S. Kim K. Cheon D. S.(2013). Seroepidemiology of predominant norovirus strains circulating in Korea by using recombinant virus-like particle antigens. Foodborne Pathog Dis 10 461–466. 10.1089/fpd.2012.1300 23627928

[R26] Tamminen K. Huhti L. Koho T. Lappalainen S. Hytönen V. P. Vesikari T. Blazevic V.(2012). A comparison of immunogenicity of norovirus GII-4 virus-like particles and P-particles. Immunology 135 89–99. 10.1111/j.1365-2567.2011.03516.x 22044070 PMC3246655

[R27] Tan M. Zhong W. Song D. Thornton S. Jiang X.(2004). *E. coli*-expressed recombinant norovirus capsid proteins maintain authentic antigenicity and receptor binding capability. J Med Virol 74 641–649. 10.1002/jmv.20228 15484274

[R28] Tan M. Jiang X.(2005). The P domain of norovirus capsid protein forms a subviral particle that binds to histo-blood group antigen receptors. J Virol 79 14017–14030. 10.1128/JVI.79.22.14017-14030.2005 16254337 PMC1280206

[R33] van Beek J. Ambert-Balay K. Botteldoorn N. Eden J. S. Fonager J. Hewitt J. Iritani N. Kroneman A. Vennema H.(2013). Indications for worldwide increased norovirus activity associated with emergence of a new variant of genotype II.4, late 2012. Euro Surveill 18 8–9. 23305715

[R34] van der Klis F. R. Mollema L. Berbers G. A. de Melker H. E. Coutinho R. A.(2009). Second national serum bank for population-based seroprevalence studies in the Netherlands. Neth J Med 67 301–308. 19687529

[R29] Verhoef L. Hewitt J. Barclay L. Ahmed S. M. Lake R. Hall A. J. Lopman B. Kroneman A. Vennema H.(2015). Norovirus genotype profiles associated with foodborne transmission, 1999–2012. Emerg Infect Dis 21 592–599. 10.3201/eid2104.141073 25811368 PMC4378480

[R30] Vinjé J.(2015). Advances in laboratory methods for detection and typing of norovirus. J Clin Microbiol 53 373–381. 10.1128/JCM.01535-14 24989606 PMC4298492

[R31] Wrammert J. Koutsonanos D. Li G. M. Edupuganti S. Sui J. Morrissey M. McCausland M. Skountzou I. Hornig M.(2011). Broadly cross-reactive antibodies dominate the human B cell response against 2009 pandemic H1N1 influenza virus infection. J Exp Med 208 181–193. 10.1084/jem.20101352 21220454 PMC3023136

