# Supplementary material for: Comparison of norovirus genogroup I, II and IV seroprevalence among children in the Netherlands, 1963, 1983 and 2006
Source: J Gen Virol. 2016 Sep;97(9):2255–64. doi: 10.1099/jgv.0.000533 (PMC5042128; doi:10.1099/jgv.0.000533)
Supplement: Supplementary File 1 [file jgv-97-2255-s001.pdf]

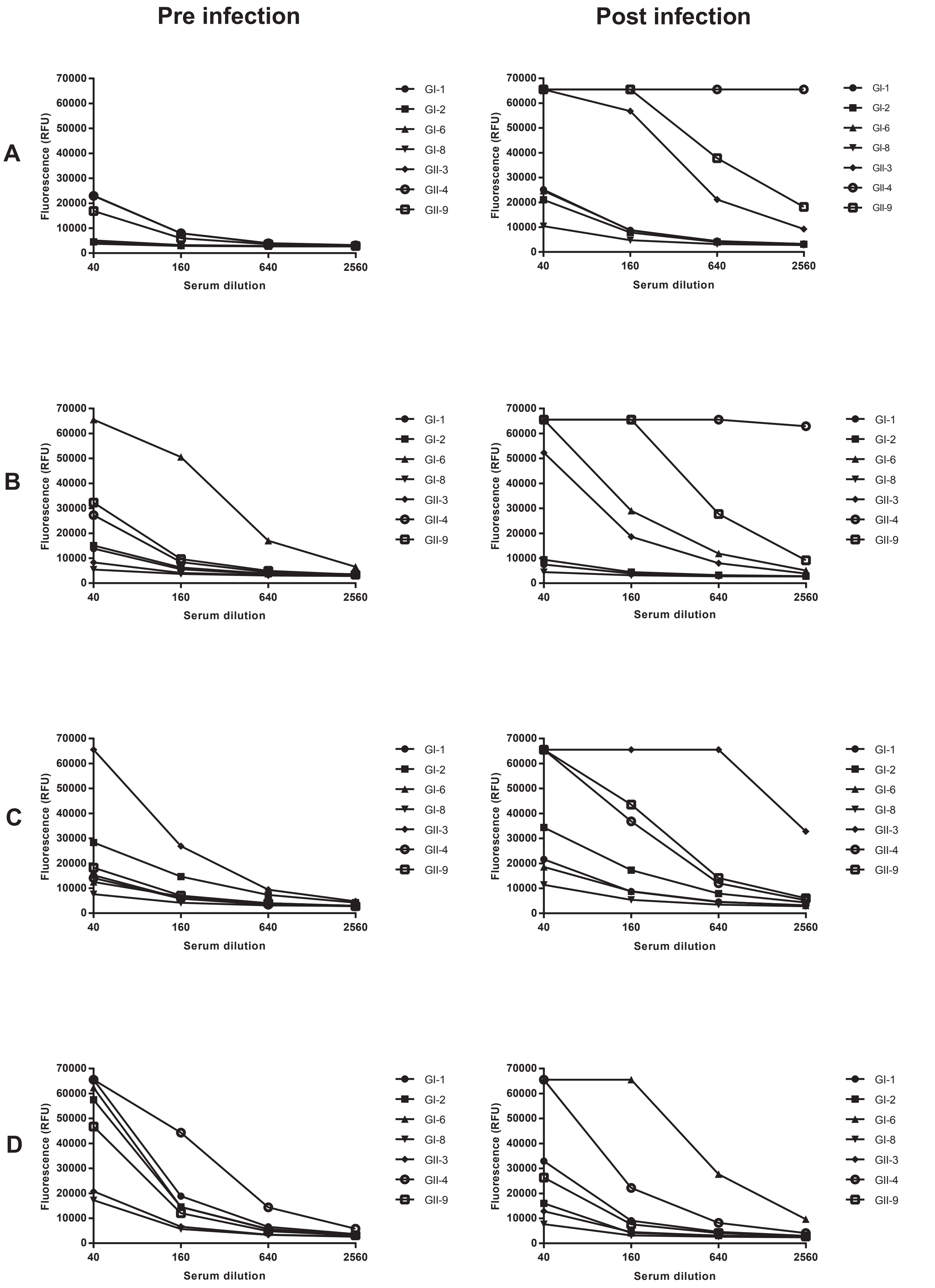

Supplemental Figure S1 Paired sera of four norovirus RT-PCR confirmed patients (ages 5, 47, 17, and 12 y respectively) infected with GII.4 Den Haag 2006b (A), GII.4 New Orleans 2009 (B), GII.3 (C), or GI.6 (D) tested against 7 different norovirus P particles on the protein array platform.

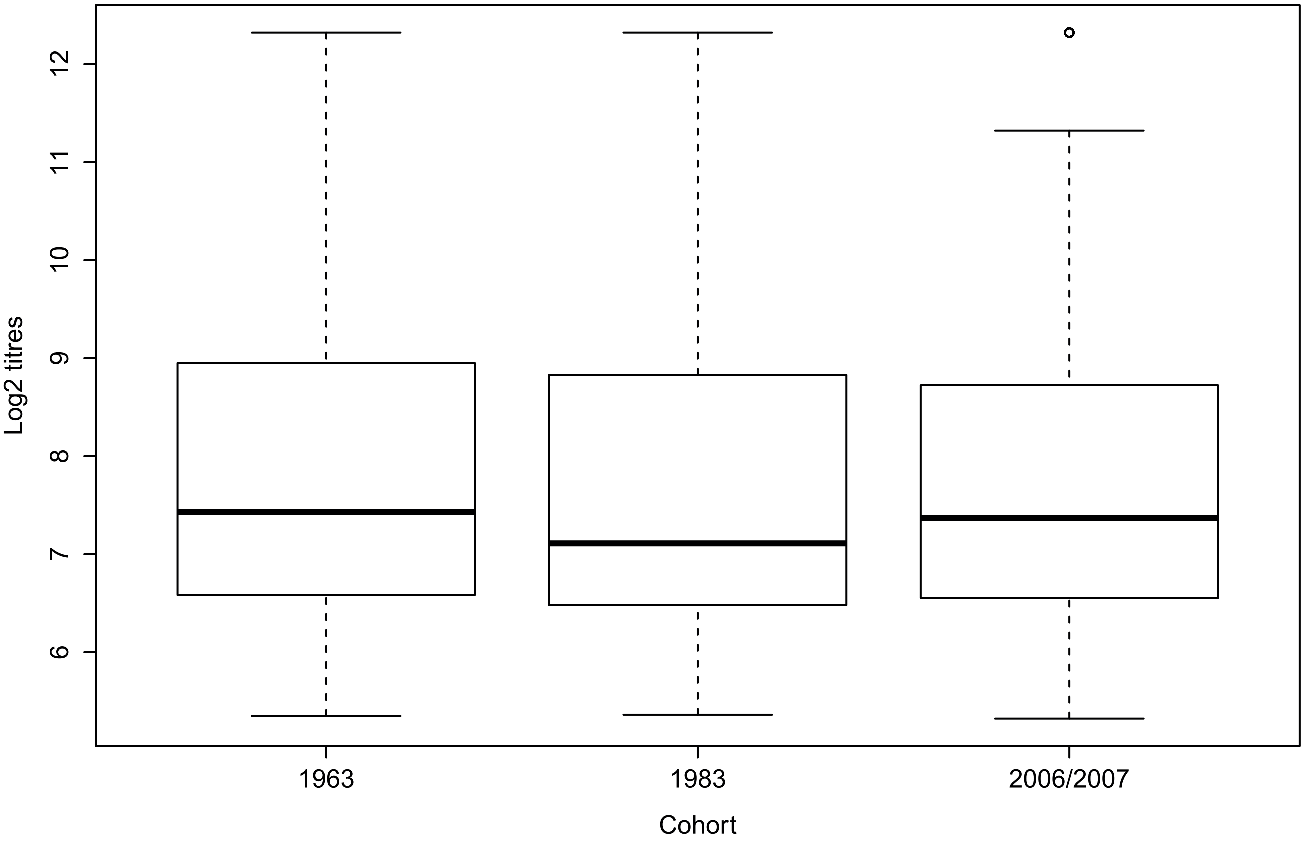

Supplemental Figure S2 Distribution of positive titres against any antigen among cohorts

Supplementary Table S1 Recombinant norovirus antigens used to test sera on the protein array platform

Genotype	Antigen type	Accession number
GI.1	P particle	M87661
GI.2	P particle	AF435807
GI.6	P particle	AF538678
GI.8	P particle	AF538679
GII.3	P particle	U22498
GII.4	P particle	AY038600
GII.9	P particle	AY038599
GIV.1	VLP	AF414427
